# Family satisfaction in the intensive care unit, a cross-sectional study from Norway

**DOI:** 10.1186/s12873-021-00412-8

**Published:** 2021-02-15

**Authors:** Randi Olsson Haave, Hilde Hammerud Bakke, Agneta Schröder

**Affiliations:** 1 Department of Health Sciences in Gjøvik, Faculty of Medicine and Health Sciences, NTNU – Norwegian University of Science and Technology, Gjøvik, Norway; 2 Innlandet Hospital Trust, Gjøvik, Norway; 3 University Health Care Research Center, Faculty of Medicine and Health, Örebro University, Örebro, Sweden

**Keywords:** Family satisfaction, FS-ICU 24, Family, Intensive care unit, Intensive care

## Abstract

**Background:**

Becoming critically ill represents not just a great upheaval for the patient in question, but also for the patient’s closest family. In recent years, there has been a change in how the quality of the public health service is measured. There is currently a focus on how patients and their families perceive the quality of treatment and care. It can be challenging for patients to evaluate their stay in an intensive care unit (ICU) due to illness and treatment. Earlier studies show that the perceptions of the family and the patient may concur. It is important, therefore, to ascertain the family’s level of satisfaction with the ICU stay.

The aim of the study was to describe how the family evaluate their satisfaction with the ICU stay. A further aim was to identify which demographic variables were associated with differences in family satisfaction.

**Method:**

The study had a cross-sectional design. A sample of 57 family members in two ICUs in Norway completed the questionnaire: Family satisfaction in the intensive care unit 24 (FS-ICU 24). Statistical analysis was conducted using the Mann-Whitney U test (U), Kruskal Wallis, Spearman rho and a performance-importance plot.

**Results:**

The results showed that families were very satisfied with a considerable portion of the ICU stay. Families were less satisfied with the information they received and the decision-making processes than with the nursing and care performed during the ICU stay. The results revealed that two demographic variables – relation to the patient and patient survival – significantly affected family satisfaction.

**Conclusion:**

Although families were very satisfied with the ICU stay, several areas were identified as having potential for improvement. The results showed that some of the family demographic variables were significant for family satisfaction. The findings are clinically relevant since the results can strengthen intensive care nurses’ knowledge when meeting the family of the intensive care patient.

## Background

Intensive care units (ICUs) are highly technological wards where critically ill patients receive treatment, nursing and care. The reasons for admission to intensive care are many and complex. The patient’s recollections of the ICU stay may consist of unclear memories and hallucinations caused by ICU delirium as well as actual events [1–3]. Studies show that patients can develop post-intensive care syndrome (PICS) as a result of the ICU stay [4, 5].Becoming critically ill represents not merely a great upheaval for the patient but also for their close family. Family members play a key role as both mediators of the intensive care patient’s needs and wishes and as a health-promoting resource that can improve patient outcomes [6, 7]. Family members’ needs and wishes are important in terms of both their role as supporters for the patients, and their own personal needs. Studies show that there may be a high prevalence among family members of depression, post-traumatic stress disorder (PTSD) or PICS-Family at the end of an ICU stay [8, 9].

Many families experience the time spent in the ICU as challenging [10, 11] and full of uncertainty regarding the intensive care patient’s condition, treatment and prognosis [12]. Family members describe the experience and the sight of the intensive care patient as well as the surroundings of the hospital bed, as frightening and unreal [13, 14]. They want to participate in patient care [7, 10] and be included in decision-making processes [12, 15]. Dodek et al. [16] and Wall et al. [17] claim that there is a potential for improvement in respect of the family’s perceptions of receiving support in the decision-making processes. Information exchanges where the ICU nurse is not present can make further communication between them and the family members more difficult [15, 18, 19]. Nevertheless, family members describe the support they receive from the ICU nurses as crucial for them in coping with the situation and understanding what is happening [12, 14].

In recent years, the patient’s perceptions of quality of care or satisfaction and the views of their family have been highlighted and used as one of several internationally recommended quality indicators for intensive care medicine [20, 21]. Donabedian [22] asserts that client satisfaction is very important as a measurement of the quality of medical services and provides information about whether health personnel have successfully responded to the client’s values and expectations. However, on account of their illness and treatment, it can be challenging for patients to evaluate the ICU stay [23]. Studies show that the patient’s and the family’s perception of an ICU stay can be the same [24–26]. In recent years, knowledge about the experiences of the patient and their family during the ICU stay has resulted in increased research on family satisfaction [6, 27, 28].

Family satisfaction with the care received by the patient during an ICU stay can be an important piece of information used in overall ICU quality enhancement, ensuring that the care provided meets both the patient’s and the family’s needs [29]. Few quantitative studies on family satisfaction following an ICU stay have hitherto been published in Norway. More knowledge is vital for both safeguarding the needs of family members and for assessing transferability between national and international research. It is also important to investigate how different demographic variables affect family members’ satisfaction ratings.

The aim of the study was to describe how family members assessed their satisfaction with the ICU stay using the Family Satisfaction in the Intensive Care Unit 24 (FS-ICU 24) questionnaire. Another aim was to identify which demographic variables were associated with differences in family satisfaction.

## Methods

### Design

The study had a cross-sectional design.

### Setting

The hospitals with the two ICUs included in the study were located in medium-sized towns in Norway with a population of just over 30,000 [30]. The two ICUs had a comparable catchment area, level of treatment, and both medical and surgical intensive care patients. The ICUs had a nurse-to-patient ratio of 1:1–2 and a senior consultant in anaesthesiology in 24-h attendance. Both ICUs had family waiting rooms that family members could use when not accompanying the ICU patient. The two ICUs had an equal number of visiting hours and a clear policy of welcoming relatives within their units. However, there were no written guidelines about caring for the relatives.

### Participants

Participants included family members, aged 18 and over, of ICU patients hospitalized for more than 48 h or, family members of patients who had died in the ICU. Other inclusion criteria were ability to read and write Norwegian and registered as the main family member (only one per patient) in the patient record. The family members were aged between 29 and 85 year with a mean age of 59.9 years and 61% were women (Table 1). 
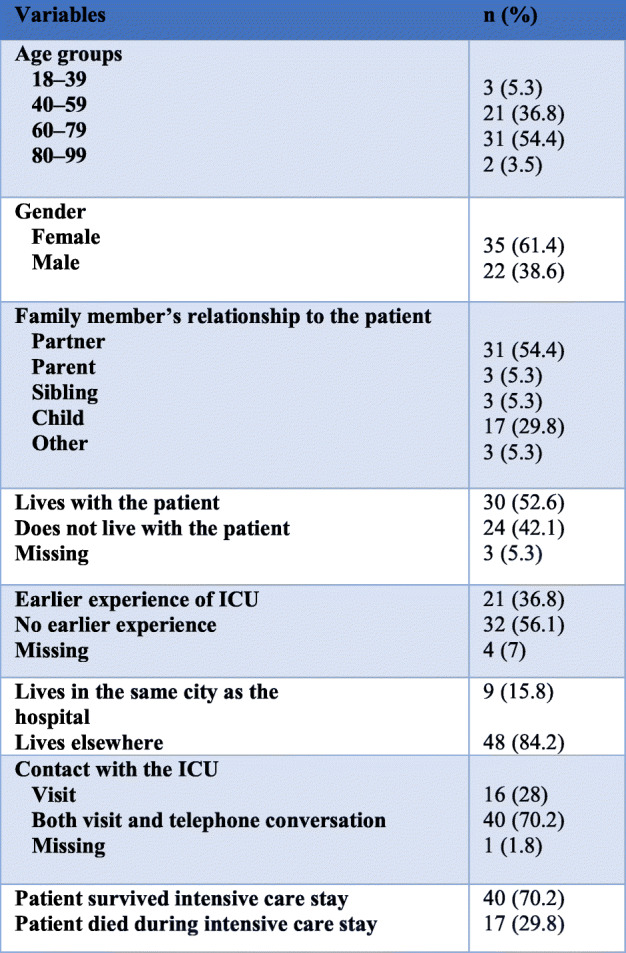

**Table 1:** Characteristics of the study group (*N* = 57)

Variables	n (%)
Age groups	
18–39	3 (5.3)
40–59	21 (36.8)
60–79	31 (54.4)
80–99	2 (3.5)
Gender	
Female	35 (61.4)
Male	22 (38.6)
Family member’s relationship to the patient	
Partner	31 (54.4)
Parent	3 (5.3)
Sibling	3 (5.3)
Child	17 (29.8)
Other	3 (5.3)
Lives with the patient	30 (52.6)
Does not live with the patient	24 (42.1)
Missing	3 (5.3)
Earlier experience of ICU	21 (36.8)
No earlier experience	32 (56.1)
Missing	4 (7)
Lives in the same city as the hospital	9 (15.8)
Lives elsewhere	48 (84.2)
Contact with the ICU	
Visit	16 (28)
Both visit and telephone conversation	40 (70.2)
Missing	1 (1.8)
Patient survived intensive care stay	40 (70.2)
Patient died during intensive care stay	17 (29.8)

### Data collection

The Norwegian Intensive Care Registry (NICR) and the Centre for Clinical Documentation and Evaluation (CCDE) carried out a Family Satisfaction in the Intensive Care Unit (FSICU) project from 2015 to 2017 in order to map out family satisfaction [31], but the data has not yet been published. The present study makes use of baseline data (October–December 2015) obtained in two of the ICUs that participated in the FSICU project. Three weeks after the ICU stay, the FS-ICU 24 questionnaire, an information letter and a prepaid envelope were sent to the main family members that met the inclusion criteria. Family members did not receive any information about the project during the ICU stay and no identifying data were included in the questionnaires so that family members could remain anonymous. Returned questionnaires were considered as written informed consent. Family members of patients with several ICU stays during the period of the study were only included once. Altogether, 165 questionnaires were distributed, of which 65 (39.4%) were returned. Eight of the questionnaires returned were excluded because they had > 30% missing values [32]. A total of 57 (34.5%) questionnaires were included in the study. The number of returned questionnaires by family members was approximately the same for both ICUs (27/47.4% versus 30/52.6%). The two groups of family members (one from each ICU) did not differ with regards demographic variables.

The FSICU project, under the auspices of NICR, was approved by the Norwegian Data Protection Authority [31]. NICR and the data protection officer at the hospitals gave written permission to use the data material in this study and considered that no other formal ethical approval was required.

### Questionnaire

The FS-ICU 24 questionnaire measures family satisfaction with the care given to the patient and their family during the ICU stay. The questionnaire is a further development of the original FS-ICU 34 version, devised by the Kingston General Hospital ICU Research Working Group [33]. The FS-ICU 24 has good psychometric properties [34, 35]. It has been translated to a number of languages and is used in many international studies [36–38]. The FS-ICU 24 includes the FS-Total score, FS-Care and FS-Decision making (DM) subscales, with responses on a Likert scale from 1 to 5, where 1 is poor and 5 is excellent [39]. In addition, the questionnaire contains demographic questions and three open-ended questions where family members can write free-text comments. The FS-ICU 24 was translated into Norwegian and back translated to English in line with the World Health Organization’s guidelines [40, 41], but has not yet undergone validity and reliability testing in Norway.

### Analysis

Data was systematized according to the procedure for recoding and scoring of FS-ICU 24 questionnaire [32]. The procedure describes recoding from Likert scale 1–5 to a new scale with score 0–100, where 0 = poor, 25 = fair, 50 = good, 75 = very good and 100 = excellent. Missing values < 30% were imputed using the individual family member’s mean value for the relevant subscale.

The Mann-Whitney U test (U) was used to examine whether there were any differences between family satisfaction on items, subscales and total score and the family’s demographic variables when there were two independent samples. In respect of two of the demographic variables – family member’s age and relationship to the patient – we chose to dichotomise the data material. The Kruskal Wallis test was used to analyse the age groups. Spearman rho was used to find the correlation coefficient between all the items in the questionnaire and overall satisfaction (FS-Total). A correlation analysis was performed with a performance-importance plot, where each individual point represented a specific item in the FS-ICU 24 questionnaire. All tests were two-tailed and the p-value p < 0.05 was regarded as statistically significant. The Statistical Package for Social Sciences (SPSS 24) was used.

## Results

### Patient satisfaction in the ICU

#### FS-care subscale

In FS-Care, family members reported on how they perceived patient treatment at the ICU and whether they themselves felt they were looked after (Table 2). The subscale showed that they were mostly very satisfied with how they and the intensive care patient were looked after. The item related to pain gained the highest mean score in terms of satisfaction. The three items – treating the patient with courtesy, respect and compassion, treating the family member with courtesy, respect and compassion and the nurses’ care for the patient – also received a high score (Table 2). The attention physicians attributed to the patient received the second lowest score whereas the lowest score was attributed to the atmosphere in the waiting room. 
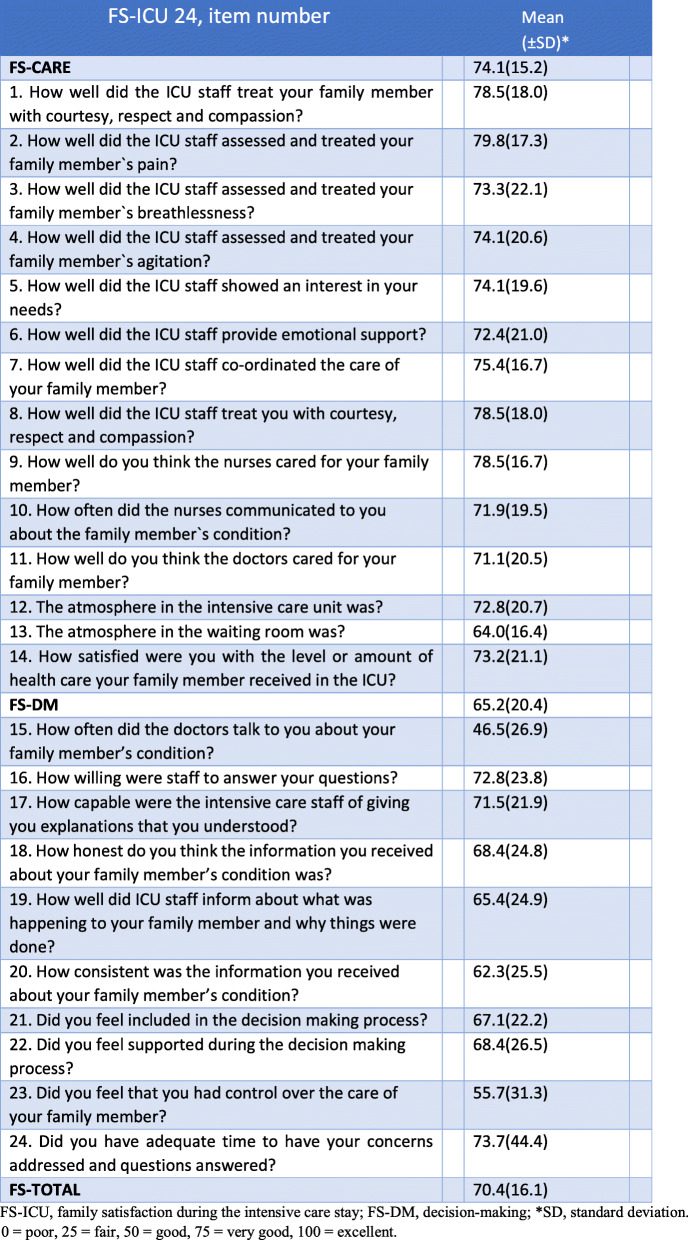

**Table 2:** Family satisfaction at item and dimension level

FS-ICU 24, item number	Mean (±SD)^a^
FS-CARE	74.1(15.2)
1. How well did the ICU staff treat your family member with courtesy, respect and compassion?	78.5(18.0)
2. How well did the ICU staff assessed and treated your family member`s pain?	79.8(17.3)
3. How well did the ICU staff assessed and treated your family member`s breathlessness?	73.3(22.1)
4. How well did the ICU staff assessed and treated your family member`s agitation?	74.1(20.6)
5. How well did the ICU staff showed an interest in your needs?	74.1(19.6)
6. How well did the ICU staff provide emotional support?	72.4(21.0)
7. How well did the ICU staff co-ordinated the care of your family member?	75.4(16.7)
8. How well did the ICU staff treat you with courtesy, respect and compassion?	78.5(18.0)
9. How well do you think the nurses cared for your family member?	78.5(16.7)
10. How often did the nurses communicated to you about the family member`s condition?	71.9(19.5)
11. How well do you think the doctors cared for your family member?	71.1(20.5)
12. The atmosphere in the intensive care unit was?	72.8(20.7)
13. The atmosphere in the waiting room was?	64.0(16.4)
14. How satisfied were you with the level or amount of health care your family member received in the ICU?	73.2(21.1)
FS-DM	65.2(20.4)
15. How often did the doctors talk to you about your family member’s condition?	46.5(26.9)
16. How willing were staff to answer your questions?	72.8(23.8)
17. How capable were the intensive care staff of giving you explanations that you understood?	71.5(21.9)
18. How honest do you think the information you received about your family member’s condition was?	68.4(24.8)
19. How well did ICU staff inform about what was happening to your family member and why things were done?	65.4(24.9)
20. How consistent was the information you received about your family member’s condition?	62.3(25.5)
21. Did you feel included in the decision making process?	67.1(22.2)
22. Did you feel supported during the decision making process?	68.4(26.5)
23. Did you feel that you had control over the care of your family member?	55.7(31.3)
24. Did you have adequate time to have your concerns addressed and questions answered?	73.7(44.4)
FS-TOTAL	70.4(16.1)

*FS-ICU* family satisfaction during the intensive care stay, *FS-DM* decision-making, ^a^*SD* standard deviation

0 = poor, 25 = fair, 50 = good, 75 = very good, 100 = excellent

#### The FS-DM subscale

The FS-DM subscale ascertains the degree of family satisfaction with the information and decision-making process throughout the ICU stay (Table 2). Family members attributed lower scores in this subscale than in that pertaining to FS-Care. The item with the highest score in FS-DM was family members’ perceptions of having sufficient time to express their concerns and receive answers to their questions. The response distribution of this item, with two alternative responses, showed that 73.7% found that they had enough time while 26.3% would have liked to have had more time to express their concerns and obtain answers to their questions (Table 2). The willingness of ICU staff to answer family member’s questions was also highly rated. The family’s perceived control over the treatment received by the patient showed the next to lowest score whereas the lowest score, both in the FS-DM and in the study as a whole, was how frequently physicians spoke to the family.

#### FS-Total

The overall satisfaction of family members (FS-Total) indicated that family satisfaction was very good (Table 2).

### Demographic variables and family satisfaction

The following demographic variables were acquired: family member’s gender, age, relationship to patient, experiences of earlier ICU stays, contact with the ICU, place of residence, and the patient’s survival or death, were analysed in order to determine whether they might be associated with differences in family satisfaction.

There was little variation amongst the different demographic variables for the FS-Care subscale. The youngest family members (18–39 years) scored highest, followed by family members living in the town where the hospital was located. Males had the lowest score.

Family satisfaction scores were lower for all demographic variables in the FS-DM subscale compared to the FS-Care subscale. The families of those who died during their ICU stay had the highest score whereas those in the youngest family member group [18–39] had the lowest. The next to lowest score was given by the partners of ICU patients.

Family who lived in the same town as the hospital had the highest overall satisfaction (FS-Total). The families of patients who died during their ICU stay had the next highest overall satisfaction score. Family members who were partners of the intensive care patient had the lowest overall satisfaction score (Table 3). 
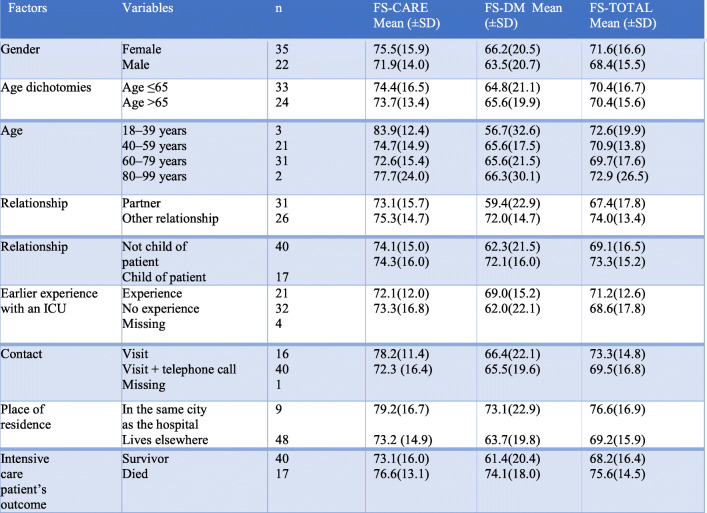

**Table 3:** Demographic factors in relation to family satisfaction (N = 57)

Factors	Variables	*n*	FS-CARE Mean (±SD)	FS-DM Mean (±SD)	FS-TOTAL Mean (±SD)
Gender	Female	35	75.5(15.9)	66.2(20.5)	71.6(16.6)
	Male	22	71.9(14.0)	63.5(20.7)	68.4(15.5)
Age dichotomies	Age ≤65	33	74.4(16.5)	64.8(21.1)	70.4(16.7)
	Age >65	24	73.7(13.4)	65.6(19.9)	70.4(15.6)
Age	18–39 years	3	83.9(12.4)	56.7(32.6)	72.6(19.9)
	40–59 years	21	74.7(14.9)	65.6(17.5)	70.9(13.8)
	60–79 years	31	72.6(15.4)	65.6(21.5)	69.7(17.6)
	80–99 years	2	77.7(24.0)	66.3(30.1)	72.9 (26.5)
Relationship	Partner	31	73.1(15.7)	59.4(22.9)	67.4(17.8)
	Other relationship	26	75.3(14.7)	72.0(14.7)	74.0(13.4)
Relationship	Not child of patient	40	74.1(15.0)	62.3(21.5)	69.1(16.5)
	Child of patient	17	74.3(16.0)	72.1(16.0)	73.3(15.2)
Earlier experience with an ICU	Experience	21	72.1(12.0)	69.0(15.2)	71.2(12.6)
	No experience	32	73.3(16.8)	62.0(22.1)	68.6(17.8)
	Missing	4			
Contact	Visit	16	78.2(11.4)	66.4(22.1)	73.3(14.8)
	Visit + telephone call	40	72.3 (16.4)	65.5(19.6)	69.5(16.8)
	Missing	1			
Place of residence	In the same city as the hospital	9	79.2(16.7)	73.1(22.9)	76.6(16.9)
	Lives elsewhere	48	73.2 (14.9)	63.7(19.8)	69.2(15.9)
Intensive care patient’s outcome	Survivor	40	73.1(16.0)	61.4(20.4)	68.2(16.4)
	Died	17	76.6(13.1)	74.1(18.0)	75.6(14.5)

No demographic variable attained statistical significance in the FS-Care subscale. The FS-DM subscale however did contain two significant findings. The first was the partner variable, attaining a statistical significance of *p* = 0.26 (Table 4) where family members in a relationship other that of partner were more satisfied (Table 3). The finding resulted in an effect size (r) of 0.296, indicating a small to medium effect (Table 4). The second variable attaining statistical significance, *p* = 0.01, was that concerning ICU survival (Table 4). Families of patients who died during their ICU stay had a higher satisfaction score in this subscale than those from families of patients who survived the ICU stay (Table 3). The effect size (r) of 0.343 indicated a medium effect (Table 4). No statistically significant differences were observed between the above demographic variables and the overall satisfaction score (FS-Total). 
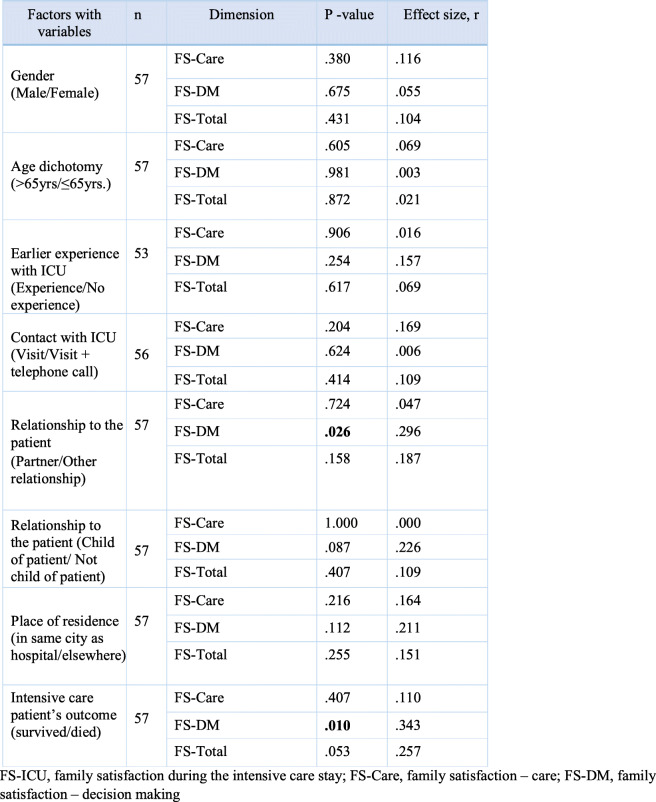

**Table 4:** Correlation between the FS-ICU 24 dimensions and demographic factors (*N* = 57)

Factors with variables	*n*	Dimension	*P* -value	Effect size, r
Gender (Male/Female)	57	FS-Care	.380	.116
		FS-DM	.675	.055
		FS-Total	.431	.104
Age dichotomy (>65yrs/≤65yrs.)	57	FS-Care	.605	.069
		FS-DM	.981	.003
		FS-Total	.872	.021
Earlier experience with ICU (Experience/No experience)	53	FS-Care	.906	.016
		FS-DM	.254	.157
		FS-Total	.617	.069
Contact with ICU (Visit/Visit + telephone call)	56	FS-Care	.204	.169
		FS-DM	.624	.006
		FS-Total	.414	.109
Relationship to the patient (Partner/Other relationship)	57	FS-Care	.724	.047
		FS-DM	**.026**	.296
		FS-Total	.158	.187
Relationship to the patient (Child of patient/ Not child of patient)	57	FS-Care	1.000	.000
		FS-DM	.087	.226
		FS-Total	.407	.109
Place of residence (in same city as hospital/elsewhere)	57	FS-Care	.216	.164
		FS-DM	.112	.211
		FS-Total	.255	.151
Intensive care patient’s outcome (survived/died)	57	FS-Care	.407	.110
		FS-DM	**.010**	.343
		FS-Total	.053	.257

*FS-ICU* family satisfaction during the intensive care stay, *FS-Care* family satisfaction – care, *FS-DM* family satisfaction – decision making

The result of the Kruskal Wallis analysis of family members’ age groups and FS-ICU 24 subscales and overall satisfaction showed that there were no significant differences.

Table 5 presented the items that showed a statistical significance in relation to the various demographic variables. Family members who were not the patient’s partner were significantly more satisfied with inclusion (*p*= .007), control (*p*= .014) and support in decision-making processes (*p*= .001) than family members who were partners. The patient’s survival or death during the ICU stay also had significance for family satisfaction. The family of intensive care patients who died during the ICU stay were significantly more satisfied with how often the physician talked to them about the patient’s condition (*p*= .003), how well they were kept informed about what was happening to the patient (*p*= .021), consistency in the information given (*p*= .024), and support in the decision-making process (*p*= .013) (Table 5). 
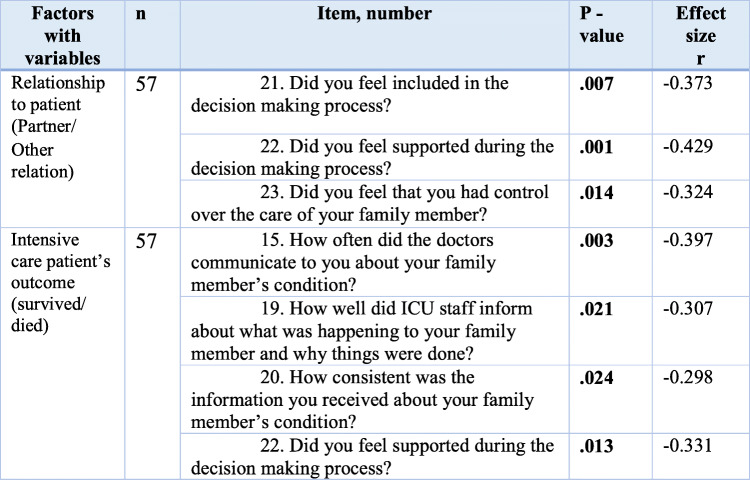

**Table 5:** Association between statistically significant items in FS-ICU 24 and demographic factors (*N* = 57)

Factors with variables	*n*	Item, number	*P* -value	Effect size r
Relationship to patient (Partner/Other relation)	57	21. Did you feel included in the decision making process?	**.007**	-0.373
		22. Did you feel supported during the decision making process?	**.001**	-0.429
		23. Did you feel that you had control over the care of your family member?	**.014**	-0.324
Intensive care patient’s outcome (survived/died)	57	15. How often did the doctors communicate to you about your family member’s condition?	**.003**	-0.397
		19. How well did ICU staff inform about what was happening to your family member and why things were done?	**.021**	-0.307
		20. How consistent was the information you received about your family member’s condition?	**.024**	-0.298
		22. Did you feel supported during the decision making process?	**.013**	-0.331

### Correlation between items in the FS-ICU 24 questionnaire and overall satisfaction

**Fig. 1:**
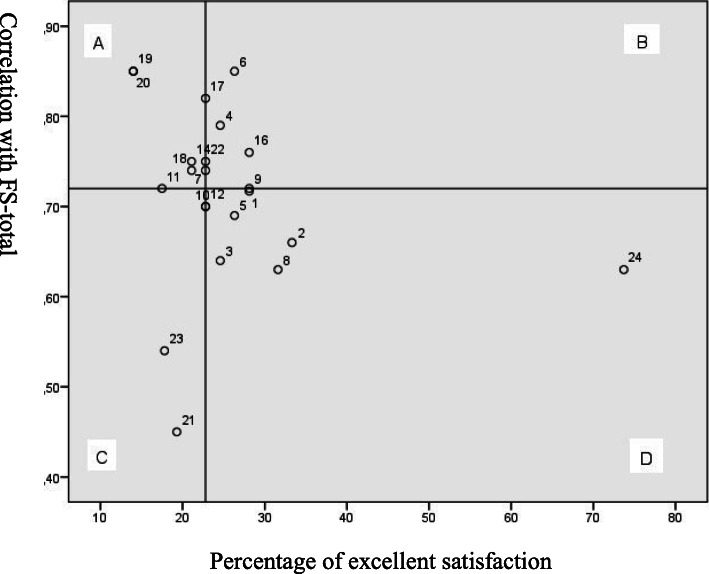
Performance-importance plot for overall satisfaction (FS-Total)

The possible association between the FS-ICU 24 items and overall satisfaction (FS-Total) was examined using a performance-importance plot (Fig. 1).

Quadrant A represents the items rated by the family members as excellent that were also highly correlated with the FS-Total. Items 19 and 20 stood out in this respect and these items concerned how well family members were informed about what was happening to the patient and the degree of consistency in the information provided by the ICU staff. For each of the items, only eight family members (14%) responded ‘excellent’. The other items in quadrant A concerned honest and understandable information, collaboration between ICU staff, the attention physicians paid, the scope of the treatment and support in decision-making.

## Discussion

Our data suggests that family members were generally satisfied with the care that was received in the ICU. Family members were equally satisfied with the nursing care and with the overall treatment (FS-Care), for both the hospitalized patient and for the family itself. The degree of satisfaction was somewhat lower in relation to the information given by ICU staff, and family member’s participation in the decision-making process (FS-DM). Our results also showed that two demographic variables, a family relationship other than that of partner and, families of patient who died during their ICU stay, were associated with a high degree of satisfaction. Two items of the FS-ICU 24 questionnaire, obtaining complete and consistent information, were highly correlated with overall satisfaction, although a low percentage of family members assessed these items as excellent.

### Family satisfaction in the ICU

#### The FS-care subscale

The FS-Care subscale showed a higher degree of satisfaction by family members than the FS-DM subscale. Earlier international studies that have used FS-ICU 24 report similar findings [36, 42–44].

Both ICUs had waiting/family rooms and family members assessed their satisfaction with the atmosphere in them as being considerably lower than for the other items of the FS-Care subscale. Similar findings have been reported in prior studies where the FS-ICU 24 questionnaire was used [43, 45, 46]. Engström and Söderberg [13] reported that family members greatly appreciated this room as it gave them the opportunity to step back while remaining close to the ICU patient. In contrast, other studies [11, 14] have reported that the time spent in the waiting room is perceived as stressful as family members would rather be with the patient. In addition, the limited facilities that these rooms provide are described in several studies in either a negative fashion or, as areas for potential improvement [18, 19, 25, 47].

Low satisfaction with either pain or agitation relief has been reported in several earlier studies where FS-ICU 24 was utilised [37, 43, 45, 47]. During an ICU stay, many patients develop delirium, which can cause agitation and confusion [48, 49]. It can be frightening and difficult for the family to see that their loved ones are in pain and that there is a change in their mental status. In contrast, our data suggests that family members rated their satisfaction with symptom relief as very good. There may be several reasons for this divergence. First, there may be country specific variations regarding symptom relief in the ICU [50] and second, an increased focus on analgosedation in Norwegian ICUs [51]. Nevertheless, Woien, Stubhaug and Bjork [52] found that practices between Norwegian ICUs vary and this may result in family members assessing symptom relief differently. Differences in symptom treatment should therefore be stressed when comparing the results of studies.

#### The FS-DM subscale

Our data suggests that family member’s satisfaction, as measured by the FS-DM subscale, was lower than that reported in earlier research using the same questionnaire in Canada [16], Norway [42], Germany [47] and the USA [53, 54].Family members assessed their satisfaction with the frequency of conversations with physicians as low. They expected to receive accurate and unfiltered information, even in the event of bad news [12, 13]. Dodek et al. [16], Lind et al. [15] and Wall et al. [17] suggested that family members had a low level of satisfaction with the frequency of conversations with physicians. However, the structure of the FS-DM subscale does not provide information as to the actual content of the conversation or, whether family members felt the need to talk to physicians more often than was the case. Whether family member’s response would have been different had the question been formulated differently, for example: ‘Did you receive adequate information from the physician?’ It is arguable whether frequent conversations always result in a higher degree of satisfaction or whether the content of and access to information from the intensive care staff are the most important. All patient situations are different and the need for information will vary. The family members’ perceptions of a lack of information may be because they did not understand or had not received the information they wanted, as described by Frivold, Dale and Slettebo [12] and Lind et al. [15]. Another reason may be that they did not remember the information they had been given due to the gravity of the situation.

Family satisfaction with the time available for getting answers to their questions and the willingness of intensive care staff to reply to these questions was rated as highest in the FS-DM subscale. The results were somewhat surprising since family members rated the frequency of conversations with physicians as the item with lowest satisfaction. This finding suggests that it may be ICU nurses who primarily answer many of the family’s questions. The family’s need for information about the intensive care patient’s condition, care and treatment is highlighted in a number of earlier studies [13, 18, 19, 47]. As reported by Agard and Harde’s [11] family members express the need to obtain information throughout the entire care pathway, including when intensive care staff have no new information to impart.

#### FS-Total

Although our results show that family members rated overall satisfaction with the ICU stay as high this rating was nevertheless lower than earlier research using FS-ICU 24 [16, 33, 35–37, 43, 44, 47, 53–55].

In research on people’s perceptions and satisfaction, it would be impossible and unrealistic to expect that everyone is completely satisfied with all the situations they experience [56]. Donabedian [57] states that quality demands can never be completely satisfied. Earlier studies also examined the term ‘complete satisfaction’ and raised the question of what the goal for family satisfaction should be [27, 37]. Donabedian [22] points out that family expectations and needs will not always be realistic and feasible, which may affect their perceptions of satisfaction. Family members’ limited knowledge about medical treatment and intensive care technical equipment may give them unrealistic expectations, which in turn may affect their satisfaction.

### Demography and family satisfaction

Our results showed that partners were less satisfied than family members with no marital relationship with the patient. Conversely, other researchers found no association between satisfaction and the relationship between patient and family member [25, 47, 58]. On the other hand Hunziker et al.’s [45] reported that family members who did not live together with the patient were more likely to be dissatisfied with the ICU stay, but their relationship with the patient was not further specified. The statistically significant results at item level concerned the role of family members in decision-making processes in the form of support, inclusion and control. Differences in family satisfaction based on relationships may be associated with family members’ varying expectations. Partners may have different wishes and needs in the decision-making process than other family members.

Our results showed that the families of patients who died expressed greater satisfaction overall and, in both subscales (FS-DM and FS-Care) used in this study. A number of international studies that have used FS-ICU 24 have investigated whether ICU survival has significance for family satisfaction, although the findings vary. The results of Dodek et al.’s [16] study showed that the family of patients who die rate their satisfaction with the decision-making process as higher. Pagnamenta et al. [36] and Wall et al. [17] found greater satisfaction in all subscales among the family members of patients who died. Other studies found no correlation between the survival of the intensive care patient and family satisfaction [43, 45, 47].

There may be several reasons why the family of patients who died during the ICU stay were more satisfied. Wall et al. [17] stated that the results do not indicate that the families of those who die received better care, but that they may indicate that healthcare personnel make an extra effort to meet family members’ wishes as death approaches. The results in this study showed that the families of those who died rated their satisfaction with the frequency of conversations with the physician and information about patient treatment significantly higher. These family members were also more satisfied with the consistency of the information provided and the support they received during the decision-making processes. The ICUs in this study did not have a standardised follow-up of relatives of patients who died during the stay. The intensive care staff in the wards nevertheless had an established tradition of meeting the needs of the family members.

### Association between the items of the FS-ICU 24 questionnaire and overall satisfaction

One of the main aims of measuring family satisfaction was to map this and be able to use the findings in quality improvement work [59]. The results of the performance-importance plot revealed several items that have a high correlation with overall family satisfaction, but which a small percentage of family members rated as excellent. There are two items in particular that stood out in the plot, both of which belonged to the FS-DM subscale. These two items concerned how well family members were informed about what was happening to the patient and the degree of consistency in the information given. Other international studies using FS-ICU 24 reported similar findings [36, 37]. Family members express frustration over the lack of continuity in the healthcare personnel around the patient, which makes it difficult for them to keep up with the treatment plans and contribute to the decision-making processes [18]. Schwarzkopf et al. [47] reported similar findings, where family members stated that there were many physicians with different opinions throughout the course of the ICU stay. Continuity among the healthcare team surrounding the patient can help to give family members accurate and consistent information about what is happening to the patient. The results of the performance-importance plot were useful in the quality improvement work at the two included ICUs as well as forming a basis for comparison for other studies that focus on how relatives' satisfaction can be strengthened.The lack of consistent information may indicate a lack of coordination in how ICU personnel provide this information to the families. The intensive care nurse plays an important role in supporting the family and imparts a sense of security [12]. Conversely, intensive care nurses can sometimes be regarded as vague and evasive when communicating the patient’s prognosis and risk, since these conversations often take place around the patient’s bed and are limited to the monitoring of vital signs [60]. Family members can perceive information from physicians as more honest and complete than information from the intensive care nurses [47]. The Norwegian intensive care nurse’s limited scope to inform family members about treatment and prognoses may be one of the reasons why family members rated consistency of information as low [61]. The results of this study show that it is vital that family are adequately informed such that they understand the information they receive about the patient’s condition, treatment and prognosis.

### Strengths and limitations

FS-ICU 24 is a well-validated and reliability-tested questionnaire that has been translated into several languages and used in many countries [35, 38]. One strength of this study is that the FS-ISU 24 questionnaire has good psychometric properties, while a weakness is that the Norwegian version of the questionnaire has not been validated and tested for reliability. The data material contains no details about the patient’s background information or the severity of the illness, which is a weakness in this study. The study has a small sample size and a predominance of female respondents. Small sample size is a well-known weakness in this field of research [36, 47, 54], and can cause biases that affect the possibility of generalization to other ICUs nationally and internationally. Nevertheless, this article is one of very few studies that present results from a Norwegian setting. Furthermore, it is important to gain knowledge about relatives' satisfaction with the ICU.

The study’s performance-importance plot clearly indicates areas where measures can be implemented to increase overall satisfaction with the ICU stay. According to Dodek et al. [59], quality enhancement is a key goal when charting family satisfaction and the plot therefore represents a strength for the study’s clinical relevance. Another strength is that the results of the plot can be compared with findings from other performance-importance plots conducted in international studies that have utilised FS-ICU 24.

## Conclusion

The results showed that families were very satisfied with a large portion of the ICU stay, but that there was a potential for improvement in relation to how well the family members were informed about what was happening to the patient and the degree of consistency in the information given by intensive care staff. The results also showed that the family members’ relationship to the intensive care patient and the intensive care patient’s outcome may be significant for the degree of satisfaction. The findings have clinical relevance since they indicate that different patient care pathways and different family/patient relationships may require different types of follow-up and involve different needs during the ICU stay. Involvement and the safeguarding of family members in an ICU entail work based on traditions, culture and knowledge. The results of the study may help to provide intensive care nurses with balanced knowledge that can increase their competence when they meet the families of intensive care patients. The results may also be of importance for hospital management and other political decision-makers in their work on quality enhancement and service development.

There is a need for more studies with a larger sample and studies to test the psychometric properties of the Norwegian version of FS-ISU 24.

## Data Availability

The datasets used and analyzed during the current study are available from the corresponding author upon reasonable request.

## Abbreviations

FS-Care: Family satisfaction care
FS-DM: Family satisfaction decision-making
FS-Total: Family satisfaction total
FSICU: Family Satisfaction in the Intensive Care Unit project
FS-ICU 24: Family Satisfaction in the intensive care unit 24
ICU: Intensive care unit
NICR: Norwegian Intensive Care Registry
PICS: Post-intensive care syndrome
PICS-Family: post-intensive care syndrome – family
PTSD: Post-traumatic stress disorder
CCDE: Centre for Clinical Documentation and Evaluation
SPSS 24: Statistical Package for Social Sciences
WHO: World Health Organization
